# Dynamics of Erythroferrone Response to Erythropoietin in Rats

**DOI:** 10.3389/fphar.2022.876573

**Published:** 2022-04-20

**Authors:** Peng Xu, Raymond S. M. Wong, Wojciech Krzyzanski, Xiaoyu Yan

**Affiliations:** ^1^ School of Pharmacy, The Chinese University of Hong Kong, Shatin, Hong Kong SAR, China; ^2^ Division of Hematology, Department of Medicine and Therapeutics, Faculty of Medicine, The Chinese University of Hong Kong, Shatin, Hong Kong SAR, China; ^3^ Department of Pharmaceutical Sciences, University at Buffalo, Buffalo, NY, United States

**Keywords:** erythroferrone (ERFE), erythropoiesis-stimulating agents (ESAs), anemia, biomarker, chronic kidney disease

## Abstract

**Background:** Erythroferrone (ERFE) is a hormone identified recently as a master regulator connecting iron homeostasis and erythropoiesis. Serum ERFE concentrations significantly increase in animals and humans with normal or impaired kidney function after receiving exogenous erythropoiesis-stimulating agents (ESAs), which suggests it might be a predictive factor for erythropoiesis. To evaluate whether ERFE is an early, sensitive biomarker for long-term erythropoietic effects of ESAs, we investigated the relationship between ERFE dynamics and time courses of major erythropoietic responses to ESA treatment.

**Methods:** Healthy rats received single dose and multiple doses (thrice a week for 2 weeks) of recombinant human erythropoietin (rHuEPO) at three dose levels (100, 450, and 1350 IU/kg) intravenously. The rHuEPO and ERFE concentrations in plasma were determined at a series of time points after dosing. Erythropoietic effects including red blood cell count and hemoglobin concentrations were continuously monitored for 24 days (single dose) or 60 days (multiple doses). The expansion of erythroblasts in bone marrow was quantified by flow cytometry analysis.

**Results:** ERFE significantly increased within a few hours and return to baseline at 24 h after rHuEPO treatment. The ERFE response was enhanced after repeated treatment, which was consistent with the observed expansion of erythroblasts in the bone marrow. In addition, the dynamics of ERFE showed double peaks at approximately 2 and 10 h after rHuEPO stimulation, and the ERFE baseline displayed a significant circadian rhythm. There was a strong positive correlation between peak values of short-term ERFE responses and the long-term hemoglobin responses.

**Conclusion:** The stimulated release of ERFE is a rapid process within 24 h. The second peak in the ERFE response to rHuEPO suggests the presence of a feedback mechanism counterregulating the ESA stimulation. The early increase of ERFE at 2 h appears to be a predictor of the hemoglobin response at 14 days after single dose of rHuEPO. Under multiple-dose regimen, the enhanced ERFE responses still correlate with the peak hemoglobin responses. The ERFE baseline also exhibits a circadian rhythm.

## Introduction

Erythroferrone (ERFE) is a newly identified hormone that plays a vital role in iron homeostasis and erythropoiesis (Kautz et al., 2014a; Srole and Ganz, 2021). ERFE is produced and secreted by erythroblasts and can suppress hepcidin transcription in the liver to regulate the hepcidin–ferroportin axis. Hepcidin binds to ferroportin, the only known iron exporter, inducing ferroportin internalization and degradation and reducing iron transport activity (Nemeth et al., 2004). By suppressing hepcidin, ERFE promotes iron mobilization from storage cells and dietary iron absorption to improve iron availability for erythropoiesis. As a master regulator connecting erythropoiesis and iron metabolism, ERFE evokes much research interests in many research fields such as thalassemia, myelodysplastic syndromes, chronic kidney disease, anti-doping, hematopoietic stem cell transplant (Kautz et al., 2015; Bondu et al., 2019; Hara et al., 2019; Ramirez Cuevas et al., 2020; Pirotte et al., 2021). Reported results demonstrated that ERFE concentrations significantly increased in animals and humans with normal or impaired kidney function after receiving exogenous erythropoiesis-stimulating agents (ESAs) (Hanudel et al., 2018; Ramirez Cuevas et al., 2020; Robach et al., 2021). These findings suggest ERFE has potential as an early biomarker for erythropoiesis induced by ESAs.

ESAs therapy represented by recombinant human erythropoietin (rHuEPO), is the major treatment for anemia in patients with chronic kidney disease (CKD) or patients receiving chemotherapy (Drüeke and Massy, 2019). This treatment has revolutionized anemia management, but ESA doses must be titrated and individualized to anemic patients to achieve target hemoglobin (HGB) concentrations, thus avoiding blood transfusion while minimizing adverse cardiovascular effects (Group KAGW, 2012). This is currently guided by HGB monitoring; however, HGB response to ESA is not very sensitive and highly delayed. The hemoglobin levels in patients receiving ESAs treatment are frequently higher or lower than the recommended target levels, and HGB concentrations often take at least 4 weeks to show any significant changes after initial ESA treatment or dose adjustment (Gaweda et al., 2014; Brier et al., 2018). In addition, it is difficult to identify ESA-resistant patients early based on HGB concentrations, which may cause harm to these patients because of inappropriate dose escalation (Unger et al., 2010). Therefore, an early, sensitive biomarker that can predict the erythropoietic response and resistance to ESA is urgently needed.

Predicting erythropoietic responses and resistance to ESA by using ERFE concentrations will enable early dose adjustments, thus improving the management of anemia and reducing the risks associated with inappropriately high doses of ESA. However, the lack of studies about ERFE dynamics would block and mislead clinical trials which aim to investigate the predictive ability of ERFE in ESA treatment. The current studies about the ERFE changes, either basic research in animals or clinical trials in healthy volunteers or patients, used sparse samples and did not fully reveal the dynamics of ERFE (Kautz et al., 2015; Honda et al., 2016; Bondu et al., 2019; Hara et al., 2019; Ramirez Cuevas et al., 2020; Pirotte et al., 2021). A study in 24 males firstly investigated the role of ERFE in humans and demonstrated that ERFE responded to a very low dose of EPO (Robach et al., 2021). But in this study, ERFE levels were measured by sparse sampling for 24, 48, or 72 h post-injection, while more information about the changes of ERFE especially within the 24-h short period after dosing was unknown. A better understanding of ERFE dynamics in response to EPO can provide useful information for study design in future research. To our knowledge, there is not any preclinical data showing the relationship between early ERFE response (within a few hours after dosing) and long-term erythropoietic effects after ESA treatment. This information is critical for demonstrating the validity of ERFE as a biomarker.

To determine the detection window of significant ERFE response and the relationship between ERFE response and long-term erythropoietic effects after ESA treatment, we thoroughly investigated ERFE dynamics and erythropoietic activities after single and multiple intravenous (IV) administrations of recombinant human erythropoietin (rHuEPO) in healthy rats.

## Materials and Methods

### Animal Preparation

The studies were conducted after approval from the Animal Ethics Committee of The Chinese University of Hong Kong (Reference Number 20-30-MIS-5). Male Sprague–Dawley (SD) rats with weights ranging from 300 to 330 g were supplied by the Laboratory Animal Services Centre. The animals were kept at ambient temperature (25°C) and relative humidity (50%) with 12/12-h light/dark cycle (two to three rats per cage) and had free access to food and water. The animals were acclimatized for 1 week before the experimental procedures.

### Immunoassay

We tested two commercially available enzyme-linked immunosorbent assay (ELISA) kits for rat plasma ERFE measurement (LS-F32186, LSBio, Seattle, WA, United States; ER1573, FineTest, Wuhan, China). Samples were collected from three rats before any interference and 4 h after 2 ml of blood loss. There was no significant difference in the performance of the two ELISA kits and the kit manufactured by FineTest was used for further studies. Drug concentrations were quantified *via* a validated ELISA method using a commercial kit (DEP00, R&D Systems Inc., Minneapolis, MN, United States). The standard curve showed linearity between 2.5 and 200 mIU/ml, and the quantification limit was 0.156 μg/L. The intra- and inter-assay precision of this kit were 1.8% and 9.9%, respectively. Rat endogenous EPO concentrations were also measured by ELISA kit (ab274398, Abcam, Waltham, MA, United States). Plasma samples with high drug concentrations were diluted using the specimen diluent within the kit.

### Hematological Measurements

An auto hematology analyzer (BC-2800Vet, Mindray Medical International Limited, Shenzhen, China) was used for hematological tests to obtain RBC count, HGB concentration, mean corpuscular hemoglobin, and platelet count. Blood samples were analyzed within 4 h after sampling.

### Animal Studies

Single- and multiple-dose studies were conducted using healthy SD rats. Recombinant human erythropoietin (rHuEPO; EPOGEN^®^ 20,000 units/ml, Amgen Inc., Thousand Oaks, CA, United States) was used and diluted using saline containing 0.25% bovine serum albumin (BSA, Sigma-Aldrich, St. Louis, MO, United States). In the single-dose study, 36 rats were randomly divided into four groups (*n* = 9), the control group receiving saline with 0.25% BSA and the treatment groups receiving a single injection of 100, 450, or 1350 IU/kg rHuEPO in the tail vein. Blood samples were collected *via* the tail vein at 5, 15, 30 min and 1, 2, 4, 8, 12, 24, 32, 48 h after injection. In the multiple-dose study, the grouping and doses were the same, but the dose regimen was three times weekly for 2 weeks. Blood samples (100–150 μl) were collected via the tail vein at 5, 30 min and 1, 2, 4, 8, 12, 24, 32, 48 h after the first and sixth doses and predose for the third to fifth doses. To minimize the effects of blood loss, blood sampling was conducted in rotation with three subsets of rats (*n* = 3) for each group in single- and multiple-dose rHuEPO studies. Before sampling, the rats were anesthetized with 5% isoflurane in an induction chamber for 2 min and maintained with 2%–2.5% isoflurane via a nosecone for approximately 2 min to collect blood and stop the bleeding. Blood samples with anticoagulants (EDTA) were centrifuged (2000 g for 10 min) to collect plasma. In the study to confirm double peaks of ERFE dynamics, blood samples were collected without rotation, but the sample volumes were strictly reduced, and only ERFE was measured. In the dedicated study to confirm the circadian rhythm of baseline ERFE, blood samples were collected from three healthy rats at 8:00, 13:00, and 18:00 on the first and second days, 16:00 and 24:00 on the third day, and 8:00 and 16:00 on the fourth day.

### Immunostaining and Flow Cytometry

Two groups (*n* = 3) of rats were sacrificed to count erythroid cells in the bone marrow before rHuEPO treatment and after five doses of 450 IU/kg rHuEPO stimulation. Single-cell suspensions from bone marrow were collected from the rat femur and then counted with trypan blue staining by an automated cell counter (Invitrogen™ Countess™ 3, Thermo Fisher Scientific, Waltham, MA, United States). Bio-conjugated anti-rat erythroid cell HIS-49 and PE-conjugated anti-rat CD71 antibodies (BD Biosciences, San Jose, CA, United States) were used for identifying erythroid cells. Flow cytometry analysis was conducted using a BD LSR Fortessa Cell Analyzer (BD Biosciences, San Jose, CA, United States). Erythroid cells were stratified into three populations (early erythroblasts, late erythroblasts, reticulocytes) based on forward scatter and CD71 levels, as previously described (Yan et al., 2013). The gating strategy is shown in Figure 4A. The absolute number of subpopulation cells was calculated by percentage × the total number of living cells in one femur.

### Data Analysis

RHuEPO pharmacokinetic parameters were calculated by a non-compartmental approach using Phoenix software (Certara, Princeton, NJ). Statistically significant differences in mean measurements at each time point were assessed using a two-tailed Student’s t-test for the same dosing regimen and one-way ANOVA between groups. The confidence level was set at α = 0.05. Data handling, statistical analyses, and graphical presentations were performed in R (R Core Team) and GraphPad Prism version 9.0.0 (GraphPad Software, La Jolla, CA, United States). The circadian rhythm analysis of ERFE baseline used a “cosinor2” R package by cosinor-based rhythmometry (Cornelissen, 2014).

## Results

### Erythroferrone Response Increased Rapidly Within Hours After Recombinant Human Erythropoietin Treatment, and the Response Was Stronger Than That in Hemoglobin

**FIGURE 1:**
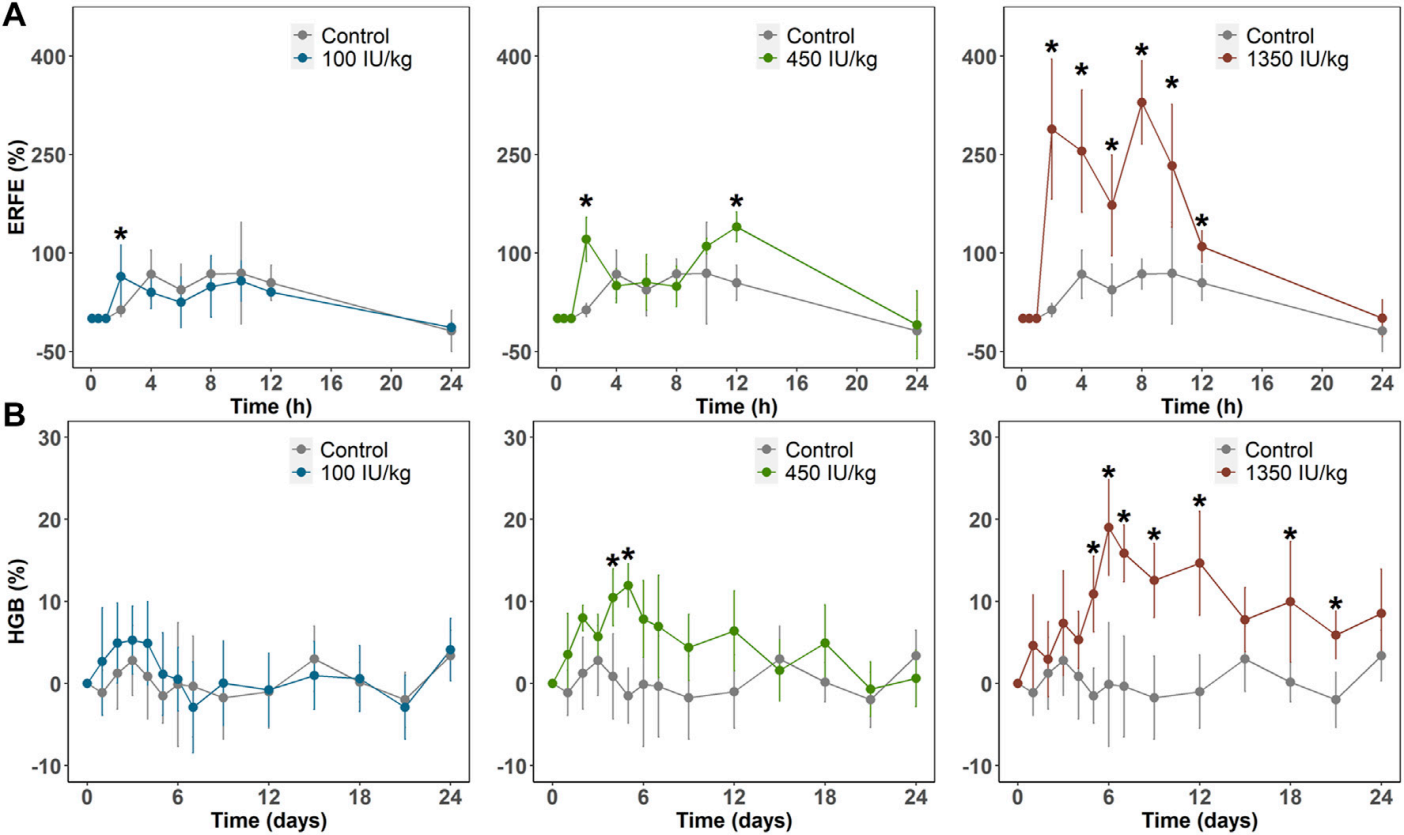
**(A)** ERFE response **(B)** and HGB response after a single IV dose (100, 450, or 1350 IU/kg) of rHuEPO. ERFE and HGB concentrations were normalized by baseline values and presented as percentage increases $\left(\frac{Value-Baseline}{Baseline}\times 100\right)$ . Data are presented as means ± SD (*n* = 3 at each time point for ERFE response; *n* = 9 for HGB response; *p < 0.05, comparison of ERFE in treatment group *versus* control group at each time point). SD, standard deviation.

In Sprague–Dawley rats weighing 300–320 g, we administered a single intravenous dose (100, 450, or 1,350 IU/kg) of rHuEPO. Blood was drawn via the tail vein to determine rHuEPO and ERFE plasma concentrations. Other pharmacodynamic (PD) markers, including red blood cell (RBC) counts and HGB concentrations, were measured. ERFE concentration exhibited a significant dose-dependent increase as early as 2 h after rHuEPO administration (Figure 1A), whereas it took 3–5 days for HGB to respond (Figure 1B). The maximum increases in ERFE concentrations were 64.1%, 139.8%, and 329.4% in the 100, 450, 1,350 IU/kg rHuEPO treatment groups, respectively, and those for HGB concentrations were 5.3%, 11.9%, and 19.0%, respectively. The ERFE response to rHuEPO was much stronger than that in HGB.

### Erythroferrone Responses Showed Double Peaks After Recombinant Human Erythropoietin Stimulation

**FIGURE 2:**
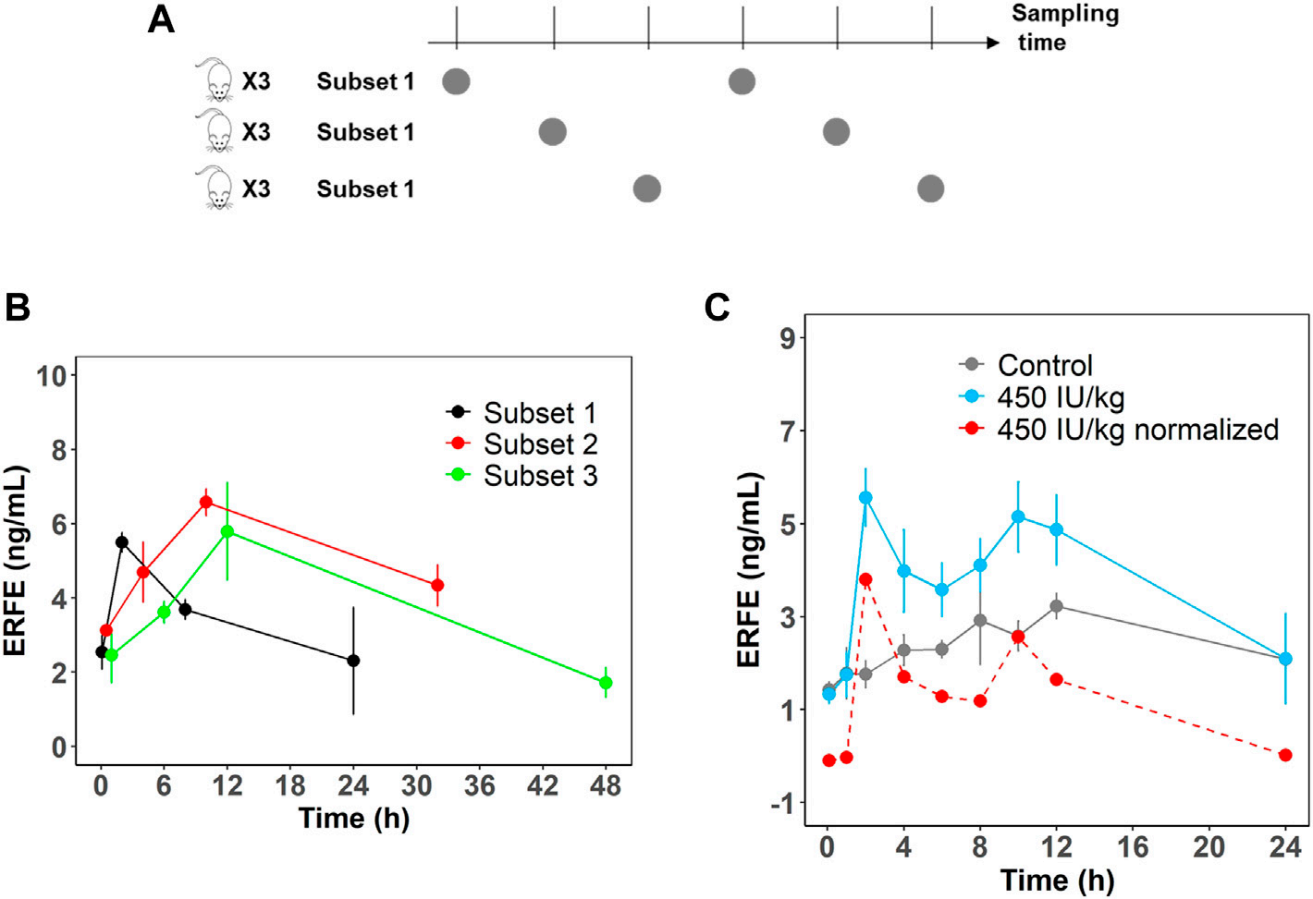
**(A)** Schematic representation of the rotation manner of sampling for the first six time points. A total of nine rats were divided into three subsets (*n* = 3). **(B)** ERFE concentrations for each subset and for combined treatment group receiving 450 IU/kg rHuEPO. **(C)** Original ERFE concentration and normalized ERFE concentrations were measured in a dedicated study after 450 IU/kg rHuEPO treatment. (*n* = 3 for each time point). Solid blue line indicates the original ERFE concentrations and was presented as means ± SD; the red dash line indicates the mean value of normalized ERFE concentrations by subtracting the mean baseline ERFE concentrations measured in the control group (solid gray line).

In the single- and multiple-dose rHuEPO studies, ERFE dynamics showed double peaks at approximately 2 and 10 h after rHuEPO stimulation (Figure 1A). We measured rat endogenous EPO concentration in the control group which did not show a significant increase (Supplementary Figure S1). This indicated the blood loss due to sampling did not trigger the increase of endogenous EPO. The double peaks might be caused by the rotating manner of blood sampling in three subsets of rats (*n* = 3 in each subset). This method was used to minimize the effects of blood loss. A total of nine rats were divided into three subsets and blood samples were not collected from the same subset (Figure 2A). ERFE displayed only one peak in each subset (Figure 2B), but when we combined three subsets, the ERFE response showed double peaks. Therefore, we conducted a dedicated study to confirm our results, by drawing blood samples in the same group without rotation to measure ERFE concentrations. The ERFE dynamics still showed double peaks (Figure 2C, blue line). We also found that ERFE expression in control rats fluctuated (Supplementary Figure S2). To exclude the possibility that the baseline ERFE fluctuations produced the double peaks, we corrected the ERFE responses by subtracting the baseline ERFE concentrations measured in the control group (Figure 2C, gray line). However, the double peaks were still present (Figure 2C, red dash line), indicating that mechanisms other than baseline fluctuation contributed to the double peaks.

### The Peak of Erythroferrone Responses Was Enhanced After Repeated Recombinant Human Erythropoietin Treatment

**FIGURE 3:**
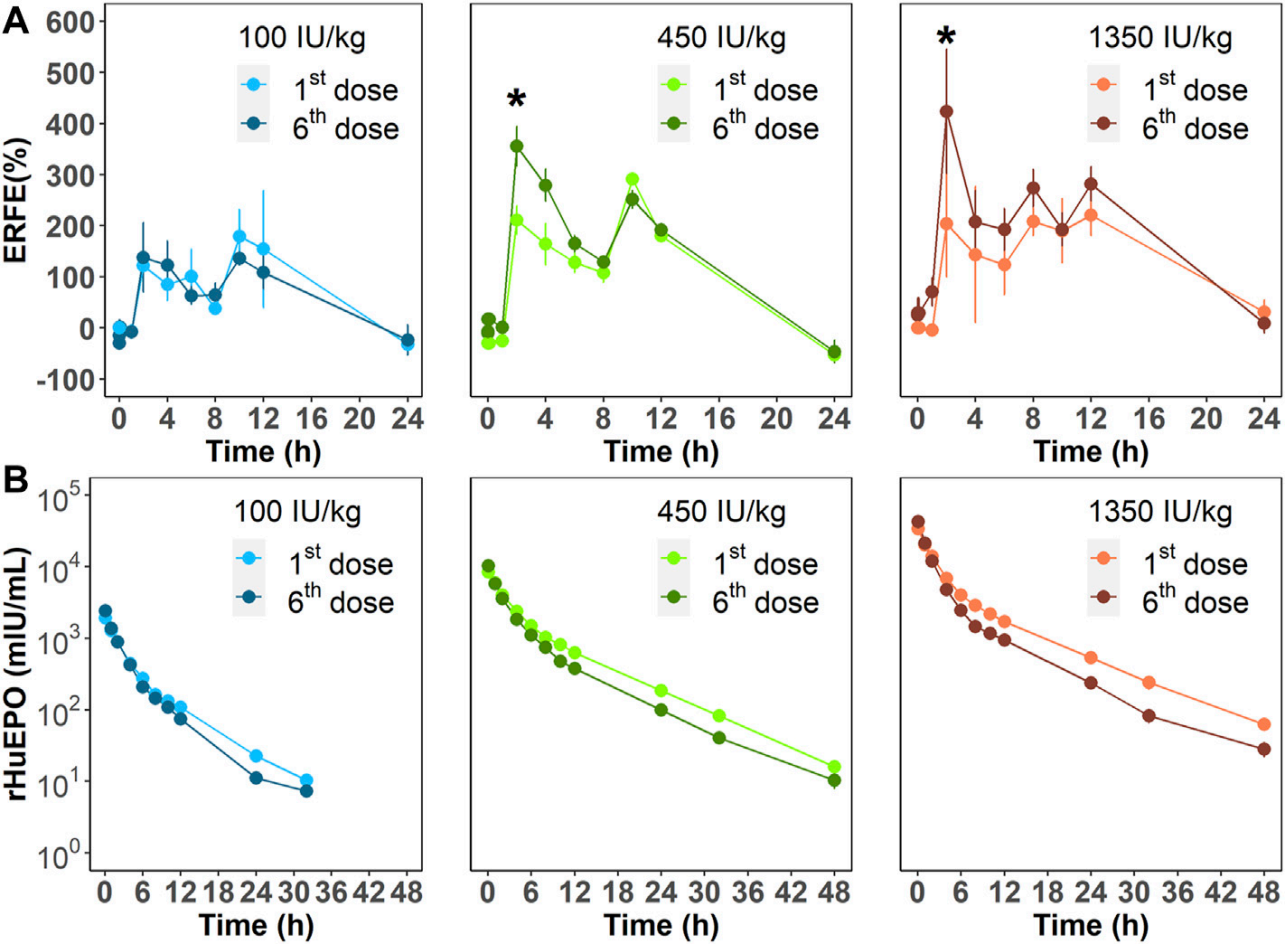
**(A)** ERFE responses after the first and sixth rHuEPO dose (100, 450, or 1350 IU/kg) and **(B)** corresponding pharmacokinetic profiles. Data are presented as means ± SD (*n* = 3 at each time point; *p < 0.05, comparison of ERFE response after the first dose *versus* the sixth dose at the indicated time point).

**FIGURE 4:**
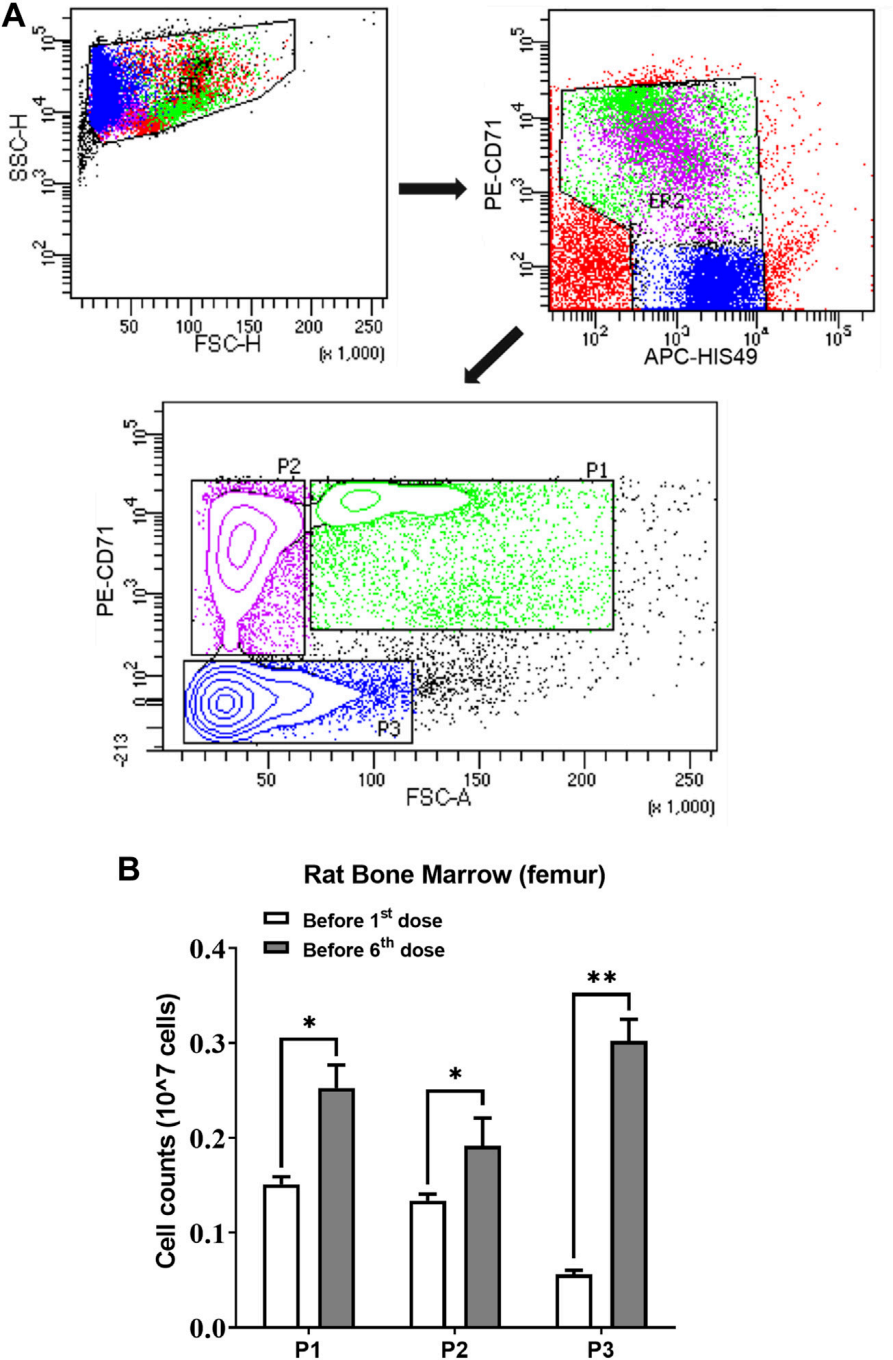
**(A)** Gating strategy of the flow cytometry to sort and quantify erythroid precursors. **(B)** Three subpopulations of erythroid cells in bone marrow quantified using flow cytometric analysis before the first and sixth rHuEPO dose (450 IU/kg; *n* = 3 in each group; *p < 0.05, **p < 0.001). P1, a mixture of proerythroblasts and basophilic, polychromatic and orthochromatic erythroblasts; P2, orthochromatic erythroblasts; P3, reticulocytes and mature red blood cells. The subpopulation cell counts were calculated by percentage × the total number of living cells in one femur.

We next investigated ERFE dynamics after multiple rHuEPO doses. Under this dosing regimen, the erythroid precursors that produce ERFE would greatly expand, which should be reflected in the ERFE dynamics. Healthy rats were treated with 100, 450, or 1350 IU/kg rHuEPO by IV administration three times weekly for 2 weeks. Blood was drawn as described above to obtain ERFE concentration time-courses after the first and sixth injections. The ERFE peak at 2 h after the last dose was significantly (for 450 and 1350 IU/kg rHuEPO group) higher than that after the first dose (Figure 3A). The drug concentrations showed no drug accumulation after multiple doses (Figure 3B); in fact, rHuEPO concentrations were reduced after repeated dosing due to receptor pool expansion and increase of receptor-mediated clearance (Supplementary Table S1), as previously reported (Yan and Krzyzanski, 2013). The apparent clearance of rHuEPO after the sixth dose was significantly higher than that after the first dose for 450 and 1350 IU/kg rHuEPO group (15.47 *vs*. 12.77, 14.72 *vs*. 12.06; *p* < 0.05). Thus, the difference in ERFE responses can be attributed to increases in erythroblast numbers, not drug concentrations. This conclusion was further supported by the erythroid precursor cell counts obtained using flow cytometry analysis. The erythroblast population was greatly expanded after five doses of rHuEPO (Figure 4B), consistent with the stronger ERFE response. Together, these findings indicate that ERFE reflects the bone marrow erythroblast proliferation induced by rHuEPO.

### Baseline Erythroferrone Concentrations Followed a Circadian Rhythm

**FIGURE 5:**
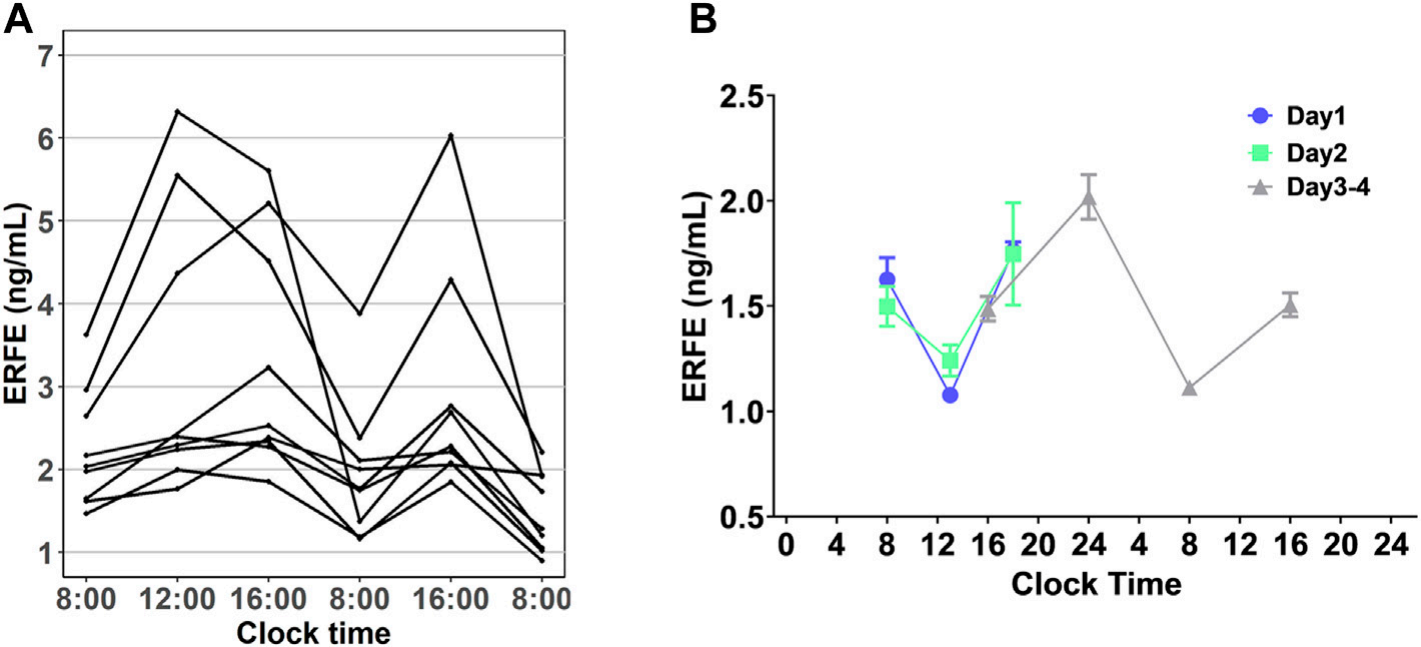
**(A)** Diurnal profile of plasma EREF in nine control rats. **(B)** Baseline ERFE concentrations measured at different time points throughout the day across four successive days in healthy rats without any treatment. Data are presented as means ± SD (*n* = 3 for each time point).

In all our experiments, we observed that the ERFE concentrations in control rats fluctuated throughout the day (Figure 5A), and it seemed there was a regularity. We hypothesized that baseline ERFE concentration fluctuations follow a circadian rhythm based on reports that the endogenous serum EPO and hepcidin concentrations show diurnal variation (Ashby et al., 2009; Pasqualetti et al., 1996). To test our hypothesis, we measured baseline ERFE concentrations three or four times daily across four successive days, according to the standard clinical approach for detecting the circadian rhythm of cortisol (Dettweiler et al., 2017). ERFE concentrations fluctuated throughout the day (Figure 5B) but showed no significant changes at the same time points across multiple days. Data were analyzed using a “cosinor” method, in which a model based on the cosine function was fitted to the data (Supplementary Figure S3). ERFE concentrations exhibited a significant (*p* = 0.011) circadian rhythm in a 24-h period (Cornelissen, 2014). The percent rhythm, i.e., the proportion of variance explained by the rhythm, was estimated to be 89% (*p* = 0.007).

### The Peak Values of Erythroferrone and Hemoglobin Responses Were Positively Correlated

**FIGURE 6:**
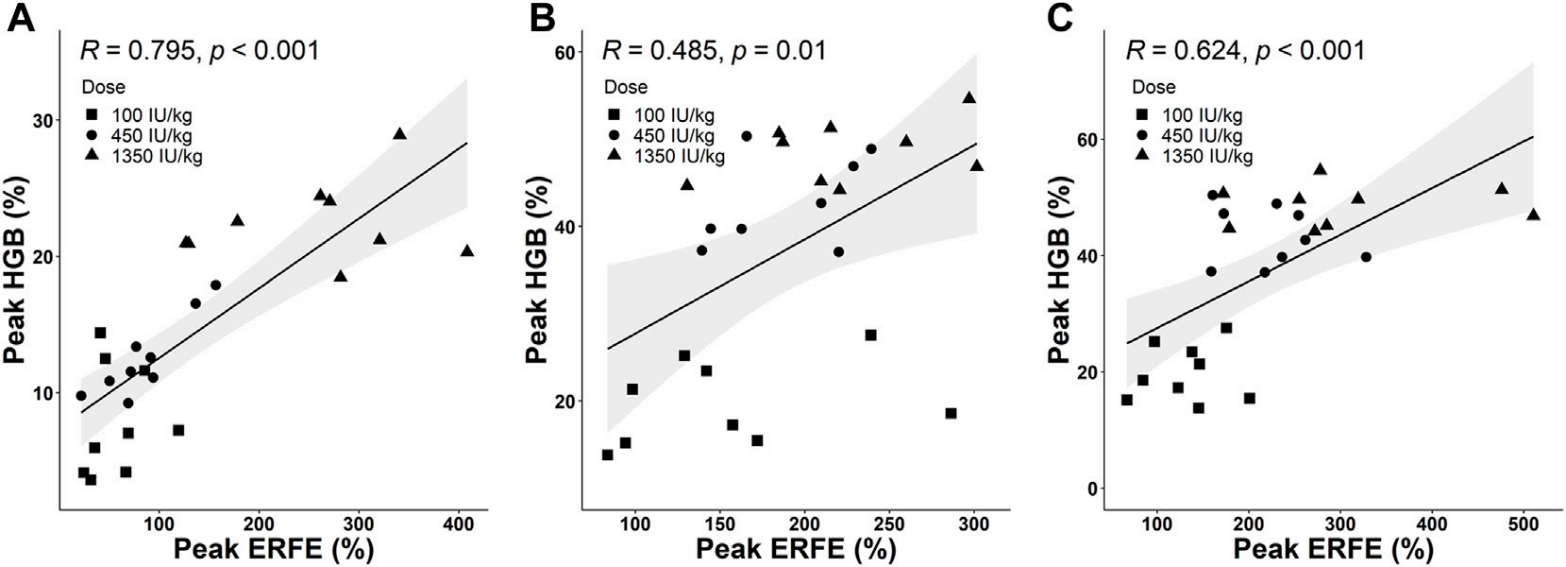
**(A)** The correlation between peak values of normalized ERFE and HGB response in the single-dose study. **(B,C)** The correlations between peak values of normalized ERFE and HGB response after the first dose **(B)** and sixth dose **(C)** in the multiple-dose study (Pearson’s method; R is the correlation coefficient). The shaded area is the 95% confidence interval.

Based on collected PD data, we detected a positive correlation between the peak values of ERFE and HGB responses. Three time-course profiles of ERFE concentrations were collected in the single- and multiple-dose studies, which were used to investigate the relationship between ERFE and HGB responses. The ERFE and HGB concentrations were normalized by the baseline values and presented as percentage changes to reduce the influence of variability. Pearson’s correlation coefficients (*R*) between ERFE and HGB increases were 0.79, 0.49, 0.62, respectively (*p* < 0.05; Figure 6). In the multiple-dose study, the correlation between peak values of ERFE and HGB was stronger after the last dose than after the first dose. Since the ERFE peak after the last dose was closer in time to the HGB peak, it should better reflect the erythropoietic response to ESA after multiple doses. In addition, the two ERFE peak values in the single-dose study were both correlated with the HGB peak values (Supplementary Figure S4).

## Discussion

Our results support that ERFE has the potential to be an early, sensitive biomarker for erythropoietic responses to rHuEPO. This is in line with the function of ERFE as a mediator in erythropoietic stress. After exogenous EPO stimulation, ERFE plays a role in the very early stage of erythropoiesis to improve iron availability by suppressing hepcidin, and then meeting the iron demand for mature RBCs production. In *Erfe* knockout mice, failure to suppress hepcidin early resulted in a delay of several days in the recovery from anemia compared to WT mice (Kautz et al., 2014b). The early increase of ERFE after EPO stimulation can be considered as the ability to mobilize iron for hemoglobin synthesis in the process of differentiation from erythroblasts to RBCs. On the other hand, our results in the multiple-dose study demonstrated that an early increase of ERFE can reflect the cell number of erythroblasts, which means ERFE can reflect how many mature RBCs will be produced. Therefore, ERFE is naturally a sensitive biomarker of erythropoiesis. Hepcidin was regarded as a tool to predict iron needs in hemodialysis patients based on its important role in regulating iron homeostasis, but research findings did not support its utility as a biomarker (Tessitore et al., 2010). This might be because of the short half-life of hepcidin and hepcidin is affected by many factors including inflammation, infection, renal clearance, iron level itself and so on (Xiao et al., 2010; Ganz and Nemeth, 2016). Current studies demonstrated that iron supplementation did not mask the ERFE increase, and muscle damage was also not a confounder (Ramirez Cuevas et al., 2020), although other factors still need to be investigated. Most importantly, the positive correlation between the peak values of ERFE and HGB provides the first evidence that ERFE could be a useful biomarker of erythropoiesis.

Our results revealed complex ERFE dynamics. The double peaks of ERFE dynamics make it challenging to select the best ERFE concentrations to predict HGB concentrations. The sampling time for ERFE should be carefully chosen because the trough concentrations between the two ERFE peaks could be misleading. Given anemic patients, especially those receiving dialysis or chemotherapy, are often in poor health, a sparse sampling strategy may be the only viable option.

The mechanism underlying the double ERFE peak is unknown. The first peak suggested that there might be an immediate release of ERFE after EPO stimulation because the first peak occurs at about 2 hours, and it may take more than 2 hours for the ERFE mRNA to increase (Kautz et al., 2014a). For the second peak, one hypothesis is that the double peaks resulted from dual-site production of ERFE by erythroblasts in the bone marrow and spleen. This hypothesis is supported by our previous work, in which differences in the erythroid precursor response to ESAs between the bone marrow and spleen were observed (Yan et al., 2013). Moreover, ERFE mRNA levels in the spleen show double peaks (Kautz et al., 2014a) in response to EPO, and increases in ESA concentrations in the spleen are delayed compared with those in the bone marrow after intravenous administration (Kato et al., 1999; Woodburn et al., 2012). Another possibility is a feedback mechanism resulting from the inhibition of hepcidin caused by ERFE. Such prolonged downregulation of hepcidin would result in an increase of the serum iron, which might temporally inhibit the release of ERFE from the erythroblasts. Administration of monoclonal antibody against hepcidin in cynomolgus monkeys yielded spikes in the serum iron (Krzyzanski et al., 2016). A transient decrease in ERFE plasma concentration might be interpreted as a second peak. This hypothesis would imply another iron-driven mechanism controlling the ERFE production that is opposite to one caused by EPO. Further experiments relating an increase in serum iron to ERFE plasma concentrations are necessary to test this hypothesis.

The circadian rhythm of baseline ERFE concentrations might contribute to the double peaks, given that it appeared to coincide with the second peak at approximately 10 h after rHuEPO stimulation (Supplementary Figure S2). However, the most likely underlying mechanism is the diurnal rhythmicity of EPO that subsequently induces the circadian oscillations of ERFE. The magnitude of ERFE circadian changes is about 0.45 ng/ml whereas the peak value corresponding to the highest rHuEPO dose is 10.19 ng/ml. This would exclude the circadian oscillations as a sole mechanism responsible for the second peak. Although we attempted to correct for the influence of circadian rhythm by subtracting the baseline ERFE concentrations measured in the control group, the circadian rhythm in the treatment groups might differ from those in the control rats. We also acknowledge that the circadian rhythms between humans and rodents are different, but it is possible this rhythm also exists in humans. This information is important for further study design in humans.

Factors such as administration route and disease status may also affect ERFE dynamics. The pharmacokinetics of ESAs after subcutaneous administration exhibit flip-flop kinetics due to slow absorption (Yan and Krzyzanski, 2013), which may delay the peak ERFE concentration and subsequently abolish the double peaks in response to ESAs. The anemia induced by chronic kidney disease or chemotherapy, may affect ERFE dynamics as impaired erythroid expansion will affect ERFE production. Nevertheless, future studies are needed to fully elucidate ERFE responses to ESAs under various conditions to determine the optimal sampling times and the number of data points needed to reasonably predict erythropoietic responses to various ESAs. One limitation of this study is that other important markers including hepcidin and iron levels are not measured, this is because the volume of samples was strictly controlled to avoid the influence of blood loss as ERFE is sensitive to hemorrhage. Subsequent studies will measure these parameters by optimizing the sampling schedule based on the ERFE dynamics.

In summary, our studies proved that the release of ERFE after EPO stimulation was a rapid process. We observed double peaks in ERFE dynamic responses to rHuEPO after IV administration that imply a possible transient negative feedback mechanism. Endogenous ERFE exhibits the circadian rhythm. The early increase of ERFE can reflect the expansion of erythroblasts and have a positive correlation with long-term erythropoietic effects of EPO. The ERFE response is more sensitive and earlier than that of HGB, making it a suitable biomarker for ESA hyporesponsiveness. Future studies involving ERFE measurement should consider more informative sampling schedules, and the diurnal variation should also be taken into account to get more convincing results. Our findings also suggest that the ESA administration route and disease status may affect ERFE dynamics. Currently, all our results were based on normal rats. In-depth research using disease animal models is needed to better understand the influence of disease on ERFE dynamic responses, and to develop ERFE as a biomarker to predict ESA response and resistance in anemic patients.

## Data Availability

The original contributions presented in the study are included in the article/Supplementary Material, further inquiries can be directed to the corresponding author.
