# Supplementary material for: Dynamics of Erythroferrone Response to Erythropoietin in Rats
Source: Front Pharmacol. 2022 Apr 20;13:876573. doi: 10.3389/fphar.2022.876573 (PMC9065252; doi:10.3389/fphar.2022.876573)
Supplement: Supplementary file 1 [file DataSheet1.docx]

**Supplement**:

**Figure S1:** Rat endogenous EPO concentrations measured in a in a control group . Data are presented as means ± SD (*n* = 3 for each time point).


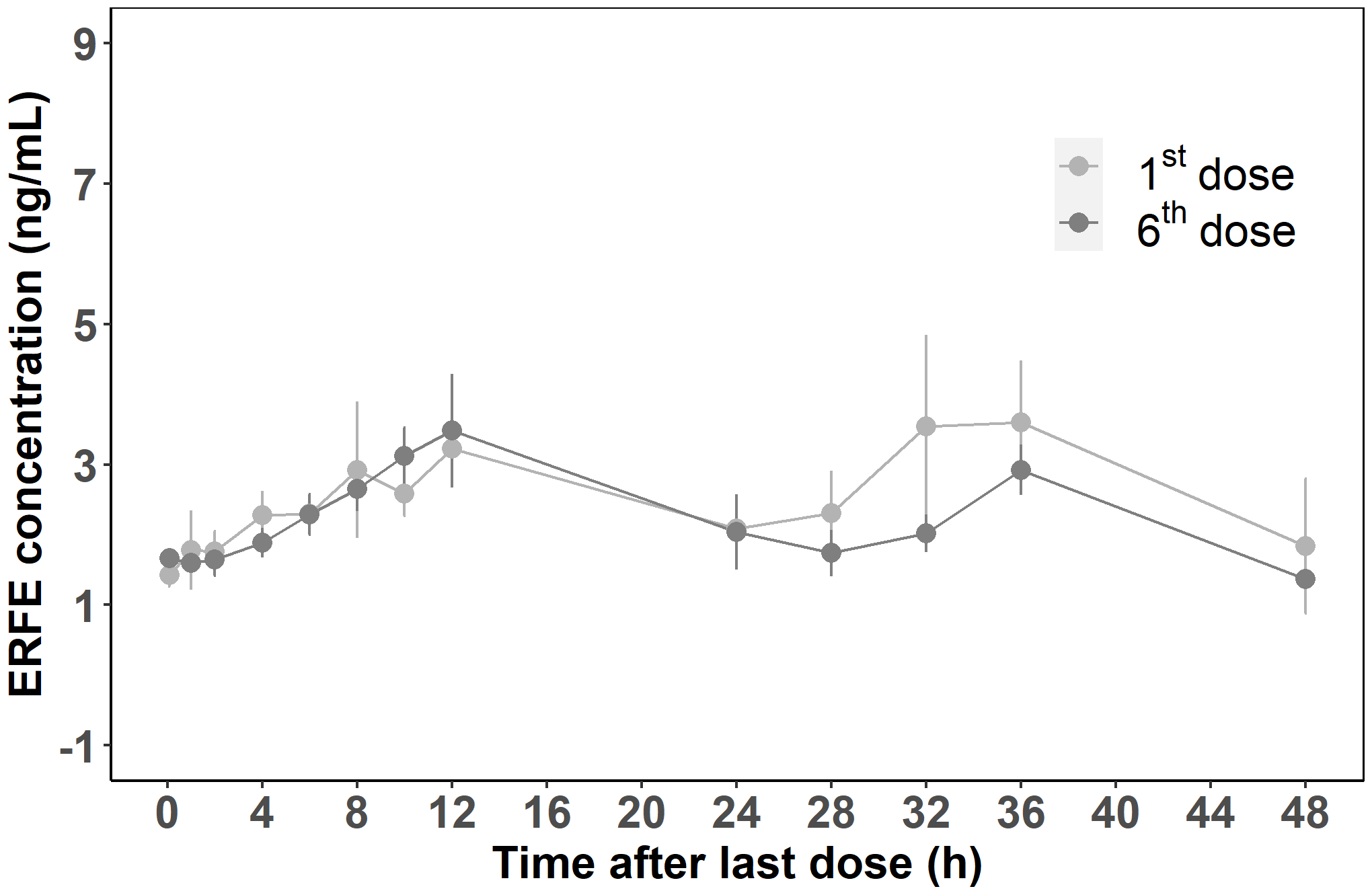


**Figure S2:** ERFE concentrations in control rats receiving saline as a vehicle in the dedicated study. Data are presented as means ± SD (*n* = 3 for each time point).


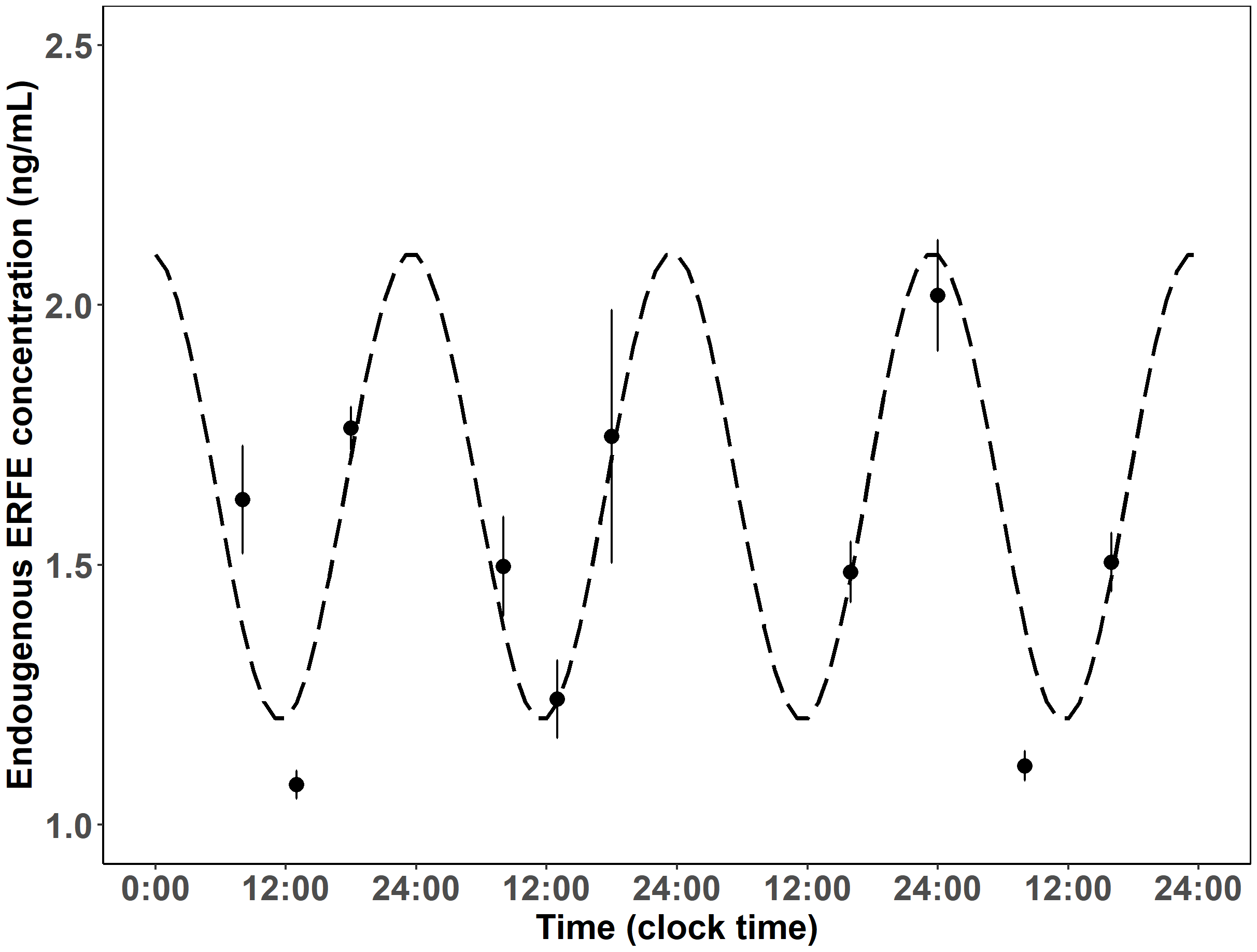


**Figure S3:** Model-estimated and observed values of ERFE showing a circadian rhythm. Points represent observed values as means ± SE (*n* = 3 for each time point). The Dash line represents the estimated values. This model is based on a single cosine function curve and fitted to data using a least-squares procedure. The regression model is written as Y(t) = M + Acos(2πt/τ – ϕ); τ is the period (duration of one cycle) and was fixed at 24 hours; M (mesor) is the mean baseline of ERFE levels and was estimated to be 1.65 ± 0.14 ng/mL; A is the amplitude and was estimated to be 0.45 ± 0.12 ng/mL; ϕ is the peak time and was estimated to be 6.15 ± 0.07 AM.


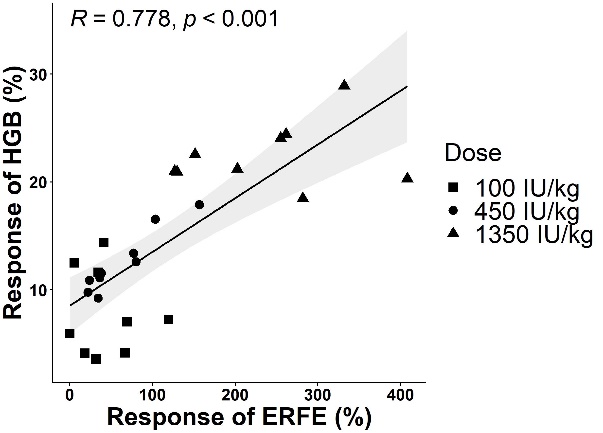

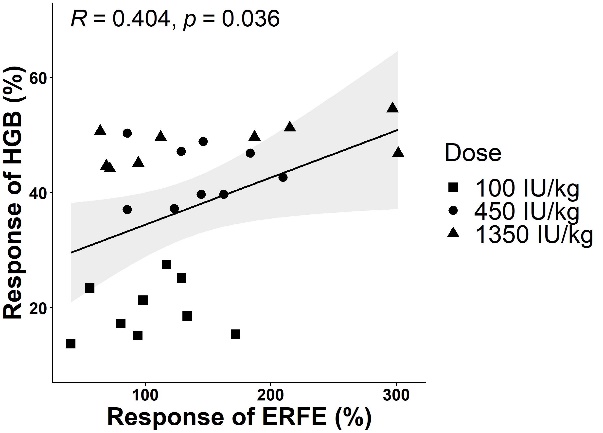

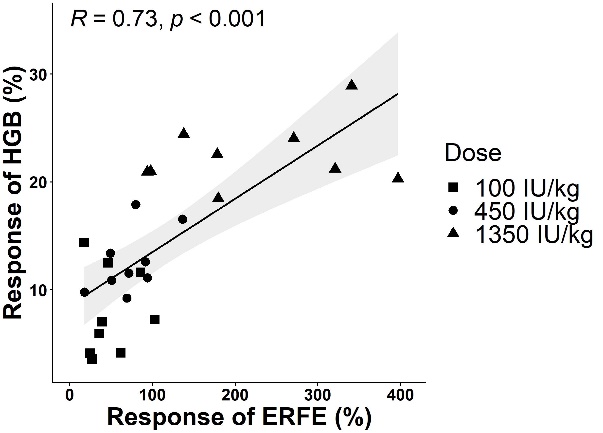


(B)

(A)


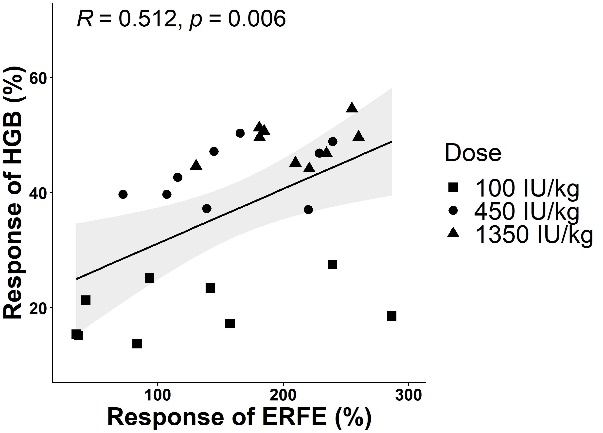


(D)

(C)


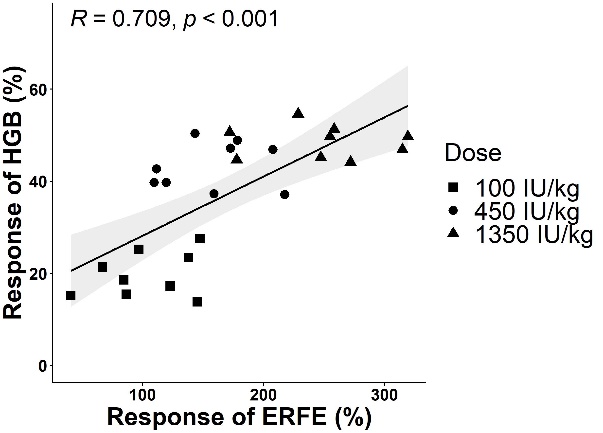


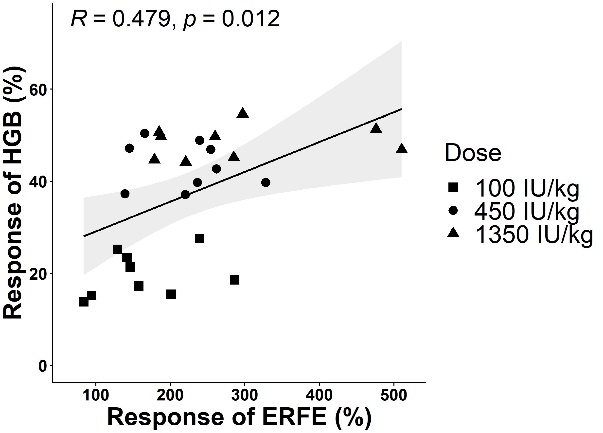


(E)

(F)

**Figure S4:** Comparison of the correlations for the two peaks of ERFE responses and peak of HGB responses in single- and multiple-dose studies. (A) The correlation between HGB peak values and the first peak values of ERFE at about 2 h after rHuEPO stimulation in the single-dose study. (B) The correlation between HGB peak values and the second peak values of ERFE at about 10 h after rHuEPO stimulation in the single-dose study. (C) The correlation between HGB peak values and the first peak values of ERFE at about 2 h after the first dose of rHuEPO in the multiple-dose study. (D) The correlation between HGB peak values and the second peak values of ERFE at about 10 h after the first dose of rHuEPO in the multiple-dose study. (E) The correlation between HGB peak values and the first peak values of ERFE at about 2 h after the sixth dose of rHuEPO in the multiple-dose study. (F) The correlation between HGB peak values and the second peak values of ERFE at about 10 h after the sixth dose of rHuEPO in the multiple-dose study.

| **Table S1. Summary noncompartmental parameters with coefficients of variations (CV%) for rHuEPO pharmacokinetics after intravenous administration of 100, 450, 1350 IU/kg TIW for 2 weeks.** | | | | | | | |
| --- | --- | --- | --- | --- | --- | --- | --- |
| Parameters (units) | Defintion | 100 IU/kg | | 450 IU/kg | | 1350 IU/kg | |
|  |  | PK1 | PK6 | PK1 | PK6 | PK1 | PK6 |
| AUC_0-48h_ (IU/mL*h) | Area under the concentration curve | 6.66 (4.5) | 6.42 (3.82) | 35.17 (6) | 29.01 (3.62) | 111.45 (4.46) | 91.5 (3.07) |
| CL (mL/h/kg) | Clearance | 14.85 (4.56) | 15.47 (3.76) | 12.77 (6.11) | 15.47 (3.7) | 12.06 (4.57) | 14.72 (3.1) |
| V_ss_ (mL/kg) | Steady-state volume of distribution | 77.92 (3.04) | 63.3 (4.09) | 83.8 (6.22) | 78.47 (2.52) | 73.38 (2.7) | 59.08 (4.39) |
| *PK1, after the first dose; PK6 after the sixth dose. | | | | | | | |
